# Comparative evaluation of root canal irrigants against *enterococcus faecalis*

**DOI:** 10.6026/973206300212337

**Published:** 2025-08-31

**Authors:** Aparajita Singh, Jitendra Lohar, Manisha Bhati, Shalini Singh, Mihir Desai, Mohit Zarekar

**Affiliations:** 1 Department of Conservative Dentistry and Endodontics, Pacific Dental College and Research Centre, Pacific Medical University, Udaipur, Rajasthan, India; 2 Department of Conservative Dentistry and Endodontics, Government Dental College and Hospital, Marwar Medical University, Jodhpur, Rajasthan, India; 3 Department of Microbiology, KMC Mangalore, Karnataka, India; 4 Department of Pediatric Dentistry, Private Pedodontic Practice, Ahilyanagar, Maharashtra, India

**Keywords:** Antibacterial efficacy, cetrimide, chlorhexidine gluconate, combination irrigants, *enterococcus faecalis*, sodium hypochlorite

## Abstract

The antibacterial efficacy of different root canal irrigants against *enterococcus faecalis*, a resilient pathogen often implicated in post-treatment infections is of interest. The effectiveness of sodium hypochlorite (NaOCl), chlorhexidine gluconate (CHX), and cetrimide was compared, both individually and in combination. The results suggest that while each irrigant has its merits, sequential use of NaOCl followed by CHX with distilled water as intermediate offers enhanced bacterial eradication. However, further research is needed to determine optimal dosages, application methods, and the most effective combinations to improve clinical outcomes and minimize post-treatment complications.

## Background:

A basic surgery in modern dentistry, root canal treatment aims to heal a tooth by removing infected or damaged pulp and sealing the root canal to prevent further bacterial infection. The great degree of efficacy of root canal therapy is defined by the ability to clean the main canal and the complicated network of lateral and auxiliary canals [1]. Especially in cases of post-treatment apical periodontitis, that is, infection at the tip of the tooth root following treatment, *enterococcus faecalis* has become one of the most persistent and virulent bacteria discovered in the root canal system. This microorganism poses a significant challenge in endodontic treatment, mainly because of its resilience and ability to survive in nutrient-deprived environments and tolerate adverse climatic conditions [2]. The encouragement of recovery of the surrounding periapical tissues in this sterile environment largely rests on root canal therapy, which aims to disinfect the canal system and completely remove the pulp tissue. Root canal irrigants are, therefore, rather crucial. Most importantly, irrigation fluids act by eliminating any residual microbes, especially the tough *enterococcus faecalis* [3]. They break down organic tissue in addition and flush debris to clear the canal. Many times, the efficacy of the disinfection process depends on the degree of the selected and used irrigant. In root canal treatment, therefore, the choice of a proper antimicrobial irrigant defines a favourable result. Throughout the years, numerous chemical substances have been proposed as suitable root canal irrigants, exhibiting varying degrees of antibacterial activity. Among the most often used irrigants are cetramide, chlorhexidine gluconate (CHX) and sodium hypochlorite (NaOCl). The popular tissue-dissolving effect and broad-spectrum antibacterial activity are characteristics of sodium hypochlorite (NaOCl) [4]. On the other hand, periapical tissues may suffer should NaOCl inadvertently cover the apex of the tooth. On the other hand, running nonstop against bacterial growth, chlorine gluconate is a powerful antibacterial agent. It has shown especially resistance against *enterococcus faecalis* and other gram-positive bacteria overall. Still, its low tissue-dissolving capacity and incompatibility with NaOCl restrict its use as a stand-by irrigant; combined with CHX, these properties cause a precipitate. Cetrimide is another quaternary ammonium compound used, sometimes in combination with other irrigants, to enhance their antibacterial action. Mostly used as a surfactant in many commercial endodontic treatments, it is highly effective against many types of infections, including *enterococcus faecalis* [5].

Considering the problems associated with single irrigation sources, combining multiple irrigants to benefit from their synergistic effects has garnered increased interest. Combining NaOCl with chlorhexidine gluconate, for example, has been proposed as a more effective method for removing tenacious bacteria, such as *enterococcus faecalis*, while reducing the negative effects of individual drugs. These irrigants taken together aim to maximize their antibacterial properties, while simultaneously decrease their specific limitations, such as toxicity or insufficient disinfection. NaOCl with chlorhexidine gluconate is hypothesised to provide a more complete technique for root canal disinfection by addressing both the bacterial biofilm and the free-floating bacteria found in the canal system [6]. These irrigants are used relatively extensively, despite the scientific community's inability to agree on the best compositions and techniques for their use. Previous studies have indicated varying degrees of efficacy in using these irrigants, either singly or in combination; some studies reveal that a single irrigant may not be sufficient to completely remove bacteria, particularly *enterococcus faecalis* [7]. Although combination irrigants may increase antibacterial activity, further analysis shows that the optimal dosages, application times, and heating methods remain unclear. More careful research is therefore urgently needed to directly assess the antibacterial action of various irrigants, both alone and in combination, if we are to inform clinical decision-making in endodontics properly [8]. Tenacity and resistance against conventional irrigants of *enterococcus faecalis* imply that root canal therapy is generally somewhat difficult. To provide more evidence of their most effective nature, this research compares the antibacterial properties of many root canal irrigants against *E. faecalis*. The results of this study could aid in improving clinical outcomes in endodontics by guiding practitioners to a greater understanding of which irrigants, or combinations thereof, are more effective in achieving total bacterial eradication. Therefore, it is of interest to maximize the long-term success of root canal treatments and minimize the emergence of post-treatment problems, ensuring the retention of pulpless teeth in a healthy and functional state.

## Methodology:

The study aimed to compare the antibacterial efficacy of various root canal irrigants against *enterococcus faecalis*, a common pathogen involved in post-treatment infections. A total of 120 extracted permanent maxillary incisor teeth were used for the experiment, which were divided into four experimental groups, each receiving a different root canal irrigant. The distribution of samples in each group was as follows: Group I received 5% NaOCl (heated to 45°C), Group II received 0.2% Chlorhexidine Gluconate (CHX), Group III received a combination of 0.2% CHX and 0.2% Cetrimide, and Group IV received a sequential combination of 5% NaOCl followed by 0.2% CHX Gluconate, with distilled water as an intermediate irrigant. Each group consisted of 30 samples (n = 30). The irrigation solutions were prepared according to the required concentrations and conditions, with NaOCl being heated to 45°C for Group I. Group III involved the combination of two substances, while Group IV involved the sequential use of NaOCl followed by CHX gluconate. The antibacterial efficacy was evaluated using both qualitative and quantitative methods. Qualitative assessment determined bacterial growth in culture media following the use of different irrigants, and results were categorized based on the presence or absence of bacterial growth, with the bacterial growth being scored using a Chi-square test for statistical significance. Quantitative assessment involved counting the Colony Forming Units (CFU) per milliliter, and each group's bacterial count was analyzed for mean, standard deviation, and standard error. The data were statistically compared using Kruskal-Wallis tests for intergroup differences, and Dunn's test was used for pairwise comparisons with Bonferroni corrections. A semi-quantitative turbidity-based evaluation was also conducted to assess bacterial growth. Bacterial growth in culture media was observed, and turbidity levels were assigned scores ranging from 0 to 3. Descriptive statistics, including mean, standard deviation, and standard error, were used to describe the central tendency and variability within each group. The Chi-Square test was applied to analyze the qualitative growth data and determine statistical significance across groups. The Kruskal-Wallis test was used for intergroup comparison, and post hoc comparisons were made using Dunn's test with Bonferroni adjustments to control for Type I errors. The Shapiro-Wilk test was used to ensure normality of data distribution, and Levene's test assessed the homogeneity of variances across the groups. The experimental protocol involved mechanically preparing the root canal systems of the extracted teeth to simulate a clinical endodontic treatment scenario. The bacterial strain *enterococcus faecalis* was introduced into the prepared root canals, and irrigation was carried out with the assigned solutions. The effectiveness of each irrigant was determined by its ability to reduce bacterial presence, measured both qualitatively (via bacterial growth or absence in culture media) and quantitatively (via CFU analysis).

## Results:

*Enterococcus faecalis* have long been implemented in persistent root canal infections and more recently has been identified as the species most commonly recovered from root canals of teeth with post-treatment disease. The primary objective of root canal therapy is the retention of the pulpless or pulpally involved tooth with its associated periapical tissues in a healthy state. Achievement of this objective requires that the pulpal spaces and contents be eliminated as sources of infection. Therefore, the introduction of an antimicrobial endodontic irrigant during root canal therapy should be given priority in the hierarchy of root canal treatment. Henceforth, the current study was done to compare the antibacterial effects of various root canal irrigants such as 5 % NaOCl, 0.2% chlorhexidine gluconate, and 0.2% cetrimide individually and combined against *E. faecalis* and gave the following result (Table 1 - see PDF). The antibacterial efficacy of four different root canal irrigants was assessed using both qualitative turbidity scoring and quantitative CFU (colony-forming unit) analysis. The sample consisted of 120 extracted permanent maxillary incisor teeth, with 30 samples in each of the four experimental groups: Group I (5% NaOCl heated to 45°C), Group II (0.2% Chlorhexidine Gluconate), Group III (0.2% Chlorhexidine Gluconate + 0.2% Cetrimide), and Group IV (5% NaOCl + 0.2% Chlorhexidine Gluconate) (Table 2 - see PDF, Figure 1 - see PDF).

The qualitative evaluation of bacterial growth was performed based on the presence or absence of growth in culture media following irrigation with various root canal irrigants. Among all groups, Group IV demonstrated the highest antibacterial effectiveness, with bacterial growth absent in 28 out of 30 samples (93.33%), and only 2 samples (6.67%) showing turbidity. Group I also exhibited strong antibacterial activity, with 24 samples (80%) showing no growth and 6 samples (20%) exhibiting bacterial presence. Group III showed moderate efficacy, with 22 samples (73.33%) growth-free and 8 samples (26.67%) demonstrating turbidity. In contrast, Group II showed the lowest efficacy, with bacterial growth absent in only 18 samples (60%), while 12 samples (40%) showed growth. The overall comparison using the Chi-Square test revealed a statistically significant difference among the groups (χ^2^ = 9.689, p = 0.021), indicating that the type of irrigant had a significant impact on inhibiting bacterial growth. The qualitative evaluation of bacterial growth was performed based on the presence or absence of growth in culture media following irrigation with various root canal irrigants. Among all groups, Group IV demonstrated the highest antibacterial effectiveness, with bacterial growth absent in 28 out of 30 samples (93.33%), and only 2 samples (6.67%) showing turbidity. Group I also exhibited strong antibacterial activity, with 24 samples (80%) showing no growth and 6 samples (20%) exhibiting bacterial presence. Group III showed moderate efficacy, with 22 samples (73.33%) growth-free and 8 samples (26.67%) demonstrating turbidity. In contrast, Group II showed the lowest efficacy, with bacterial growth absent in only 18 samples (60%), while 12 samples (40%) showed growth. The overall comparison using the Chi-Square test yielded a statistically significant difference among the groups (χ^2^ = 9.689, p = 0.021), confirming that the type of irrigant had a significant impact on the inhibition of bacterial growth. Scores 1 and 2 in contrast, Group II demonstrated the least favorable performance, with only 14 samples (46.7%) at Score 0 and some samples reaching Score 3, suggesting persistent bacterial growth. The overall Chi-square test revealed a statistically significant difference among the groups (χ^2^ = 19.431, p = 0.022), confirming that the effectiveness of the irrigants in reducing bacterial turbidity varied significantly. These results reinforce the superior performance of combined irrigants, especially the synergistic action of NaOCl and Chlorhexidine, in effectively suppressing *enterococcus faecalis* growth within root canals (Table 3 - see PDF).

Quantitative analysis of bacterial growth was conducted by evaluating the mean colony- forming units per milliliter (CFU/mL) in each group following irrigation. Group IV, which received 5% NaOCl combined with 0.2% Chlorhexidine Gluconate, demonstrated the lowest bacterial count, with a mean CFU of 0.9 x 10^3^, a standard deviation (SD) of 0.30 x 10^3^, and a standard error of the mean (SEM) of 0.055 x 10^3^, indicating superior antibacterial efficacy. Group I also showed strong antibacterial activity, with a mean CFU of 1.5 x 10^3^, SD of 0.40 x 10^3^, and SEM of 0.073 x 10^3^. Moderate antibacterial effectiveness was observed in Group III, with a mean CFU of 2.2 x 10^3^, SD of 0.50 x 10^3^, and SEM of 0.091 x 10^3^. Group II recorded the highest bacterial growth, with a mean CFU of 4.1 x 10^3^, SD of 0.80 x 10^3^, and SEM of 0.146 x 10^3^, suggesting it was the least effective irrigant among those tested. Post hoc intergroup comparisons using Dunn's test with Bonferroni correction further elucidated the statistical significance of differences between groups. A highly significant difference was found between Group II and Group IV (adjusted p < 0.001), confirming that the combination of NaOCl and Chlorhexidine was significantly more effective than Chlorhexidine alone. Significant differences were also noted between Group I and Group II (p = 0.002), Group I and Group III (p = 0.041), Group II and Group III (p = 0.013), and Group III and Group IV (p = 0.022). However, the comparison between Group I and Group IV did not reach statistical significance (p = 0.058), suggesting comparable efficacy between heated NaOCl alone and the combined irrigant, though the latter showed slightly better numerical outcomes. These quantitative findings complement the qualitative observations and further support the conclusion that the combination of 5% NaOCl and 0.2% Chlorhexidine Gluconate is the most effective irrigant in eliminating *enterococcus faecalis* from root canals.

## Discussion:

The findings of this study provides a comprehensive comparison with previous research on the antibacterial efficacy of root canal irrigants, particularly against *enterococcus faecalis*, and reveal important insights into the effectiveness of various combinations of irrigants. Sodium hypochlorite (NaOCl), known for its broad-spectrum antibacterial properties and tissue-dissolving ability, has been extensively studied and continues to be a cornerstone of root canal therapy. This study aligns with earlier studies, such as those by K Ashofteh *et al.* (2014) [9], which also emphasize NaOCl's effectiveness in eliminating bacterial biofilms, including *enterococcus faecalis*. However, the current study highlights a concern raised by Aviv *et al.* (2024) [10], where NaOCl's extravasation beyond the apex can cause significant periapical tissue damage, an issue that limits its use in clinical practice. In contrast, chlorhexidine gluconate (CHX), known for its strong antibacterial action against *enterococcus faecalis* and other gram-positive bacteria, is widely considered a key irrigant in root canal therapy. This study supports earlier research by Mohammadi *et al.* (2008) [11], which found that CHX is particularly effective against *enterococcus faecalis*, but also confirms its limitation in terms of tissue-dissolving capacity. The current study adds nuance by discussing the incompatibility of NaOCl and CHX when used together, which leads to the formation of a precipitate -a concern highlighted in studies such as those by Basrani *et al.* (2007) [12]. The negative interaction between NaOCl and CHX has been well-documented, and this study further solidifies the recommendation to avoid their simultaneous use in clinical settings. The study also notes that while CHX is highly effective in bacterial eradication, its lack of tissue-dissolving properties means it cannot fully replace NaOCl, highlighting the need for strategic combinations. Cetrimide, a surfactant and quaternary ammonium compound, is often used in combination with other irrigants to enhance antibacterial activity. The current study aligns with the findings of Mohammadi *et al.* (2008) [11], which discussed the role of cetrimide in boosting the efficacy of NaOCl and CHX. It found that cetrimide is effective against *enterococcus faecalis* and other pathogens, supporting its inclusion in combination protocols. The effectiveness of cetrimide as a standalone irrigant is less emphasized in this study compared to NaOCl and CHX. Still, its combination with other agents appears to provide a synergistic effect, as noted in previous research. This synergistic approach is consistent with findings by Sabu *et al.* (2023) [13], who observed that combining NaOCl with cetrimide improved bacterial eradication compared to using NaOCl alone, but the current study introduces the idea that more research is needed to identify optimal combinations and concentrations. The concept of combining NaOCl and CHX for improved disinfection has gained traction in the literature, and this study supports that notion, suggesting that such combinations could provide a more complete approach to root canal disinfection by targeting both biofilms and free-floating bacteria. However, the study also emphasizes the challenges of using these two agents together, given the potential for precipitate formation. Previous studies, including those by Al-Sada *et al.* (2024) [14] have highlighted that while combining NaOCl and CHX may theoretically enhance antibacterial efficacy, the formation of a precipitate is a significant limitation. The current study's finding that NaOCl and CHX combinations may improve antibacterial action is consistent with research from Luddin *et al.* [15], who noted that such combinations might offer better results than using either agent in isolation. However, the study also draws attention to the necessity of further research to optimize the dosages, application times, and specific methods to maximize the efficacy of these combinations, an area in which previous studies have also called for further investigation. The persistence of *enterococcus faecalis* in the root canal system, as emphasized in this study, is a well-documented phenomenon. Studies by Sebbane *et al.* (2024) [16] have repeatedly demonstrated the bacterium's resilience, particularly its ability to form biofilms and its resistance to conventional disinfectants like NaOCl and CHX. This study extends this understanding by emphasizing that no single irrigant is sufficient to eradicate *enterococcus faecalis* completely. The findings highlight that while NaOCl, CHX, and cetrimide each possess significant antibacterial properties, they must be used in combination to address the full spectrum of bacterial types and biofilm formations present in the root canal system. This finding aligns with previous research by Mohammadi *et al.* (2017), which recommended combining NaOCl with other agents to maximize disinfection efficacy. The study by Priya *et al.* (2024) [17] reinforces the importance of a multi-agent approach, noting that while NaOCl remains a cornerstone of root canal therapy, its combination with other agents like CHX and cetrimide enhances antibacterial efficacy. Priya *et al.* further discusses how these combinations can help address the limitations of individual agents, providing a more comprehensive solution to the biofilm formation and resistance issues commonly associated with *enterococcus faecalis*. This aligns with our findings, where we noted that using NaOCl and CHX together, despite the risk of precipitate formation, offers a better antibacterial outcome than using each alone. Furthermore, the research by Celikel *et al.* (2025) [18] explores the clinical application of these irrigants in pediatric dentistry, highlighting the challenges in younger populations where root canal treatment often faces issues of incomplete disinfection. They emphasize that while CHX is effective against gram-positive bacteria like *enterococcus faecalis*; its inability to dissolve tissue limits its clinical utility when used alone. This observation complements our findings that although CHX has superior antibacterial properties, it cannot replace NaOCl in terms of tissue dissolution. This synergy between NaOCl and CHX is crucial for comprehensive root canal disinfection. Lastly, the study by Ghorbanzadeh *et al.* (2018) [19] provides a broader context for understanding the role of alternative disinfection methods, such as photodynamic therapy, in complementing traditional irrigation protocols. Their study suggests that innovative techniques could offer synergistic effects, enhancing bacterial eradication in difficult-to-treat cases. While not a direct comparison to our study, their work encourages further exploration of combination therapies, reinforcing the need for a multifaceted approach to root canal disinfection.

## Conclusion:

The importance of selecting effective root canal irrigants to combat *enterococcus faecalis*, emphasizing the antibacterial properties of sodium hypochlorite (NaOCl), chlorhexidine gluconate (CHX) and cetrimide. It further underscores the potential of combining these agents to improve bacterial eradication, although the optimal dosages and application methods remain uncertain. Ultimately, the study provides valuable insights into improving clinical outcomes by guiding the choice of irrigants and their combinations for more effective root canal disinfection.

## References

[1] Wong J (2021). Front Oral Health..

[2] Deng Z (2023). J Oral Microbiol..

[3] Gomes BPFA (2023). Braz Dent J..

[4] Drews DJ (2023). Antibiotics (Basel)..

[5] Yared G, Al Asmar Ramli G (2020). Cureus..

[6] Mohammadi Z (2017). Open Dent J..

[7] Boutsioukis C (2022). Int Endod J..

[8] Iqbal A (2012). Int J Health Sci (Qassim)..

[9] Ashofteh K (2014). Iran J Microbiol..

[10] Aviv S (2024). Clin Oral Investig..

[11] Mohammadi Z (2008). Iran Endod J..

[12] Basrani BR (2007). J Endod..

[13] Sabu N (2022). J Pharm Bioallied Sci..

[14] Al-Sada HA (2024). Int J Dent..

[15] Luddin N (2013). J Conserv Dent..

[16] Sebbane N (2024). Microorganisms..

[17] Priya NS (2024). Int J Acad Med Pharm..

[18] Celikel P (2025). Journal of Clinical Pediatric Dentistry..

[19] Ghorbanzadeh A (2018). Photodiagnosis Photodyn Ther..

